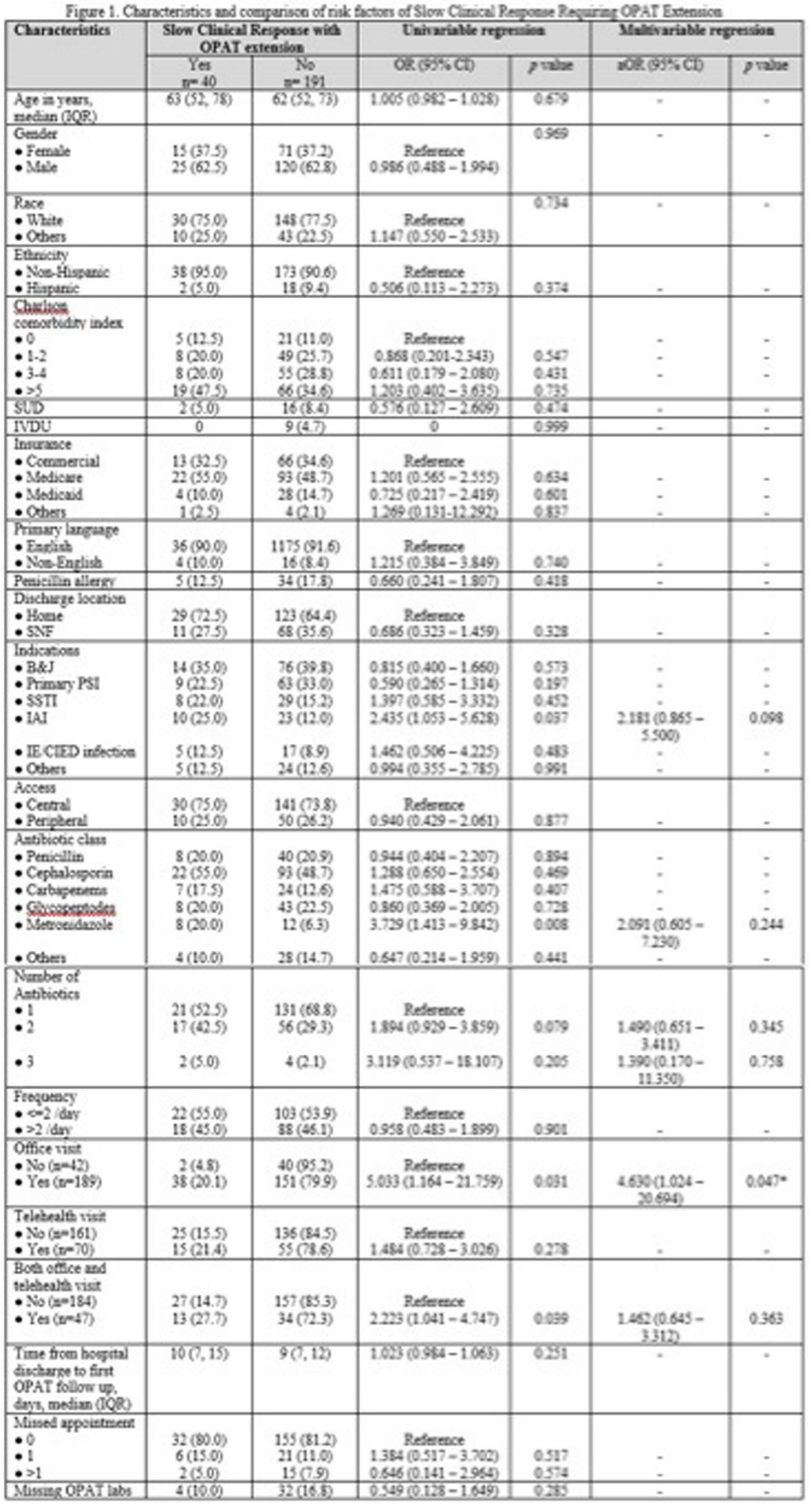# Evaluation of Predictors Associated with Slow Clinical Response with Extension of Outpatient Parenteral Antimicrobial Therapy

**DOI:** 10.1017/ash.2024.104

**Published:** 2024-09-16

**Authors:** Kristen McSweeney, Fang Yu Liu, Rachel Erdil, Majd Alsoubani, Tine Vindenes, Shira Doron, Kap Sum Foong

**Affiliations:** Tufts Medical Center; TUSM; Tufts Medicine; Tufts University School of Medicine

## Abstract

**Background:** Outpatient parenteral antimicrobial therapy (OPAT) provides a safe and effective alternative to prolonged hospitalization for patients with infectious diseases requiring elongated antimicrobial therapy. One study found that 35.6% of OPAT episodes met the composite definition for treatment failure, with unplanned extension of OPAT as the most common reason for treatment failure. Our study sought to identify factors predicting higher likelihood of extension of OPAT due to slow clinical response to treatment and determine how therapy extension relates to complications. **Method:** This retrospective cohort study included all patients aged ≥18 years discharged on OPAT between April 2022 and October 2022. Demographic, treatment, outcome, and complications data were extracted through chart review. The primary outcome was the proportion and predictors of OPAT extension due to slow clinical response to treatment. The secondary outcomes were OPAT complication rate, 30-day ED visit and 30-day readmission rates related to OPAT complications. We used univariable and multivariable logistic regression models for the primary outcome of slow clinical response requiring OPAT extension. Variables with p < 0.1 in the univariable analyses were included in the multivariable model. **Result:** 231 patients received OPAT during the six-month study. Among them, 40 (17.3%) patients required an extension of therapy. In univariable analysis, patients who had slow clinical response requiring extension of OPAT were more likely to have intraabdominal infection (odds ratio [OR], 2.435; 95% confidence interval[CI], 1.053–5.628), receipt of metronidazole (OR, 3.729; 95% CI, 1.413–9.842), and were more likely to be followed up through office visit (OR, 5.033; 95%CI, 1.164–21.759) or combination of office visit and telemedicine (OR, 2.223; 95%CI 1.041–4.747). Other variable comparisons are detailed in Figure 1. In the multivariable regression analysis, the independent predictor associated with extended of OPAT was follow-up via office visit (adjusted OR, 4.630; 95% CI, 1.024-20.694). Rates of complications related to intravenous access and antibiotic were similar between patients with and without extension; 15% vs. 11% (p=0.430) and 7.5% vs. 7.3% (p=1.000), respectively. There were no significant differences in 30-day ED visits and readmission rates between the 2 groups: 7.5% vs. 5.8%(p=0.715) and 12.5% vs. 7.3% (p=0.338). **Conclusion:** Our study highlights patient’s office visit follow-up is associated with the OPAT extension due to slow clinical response. However, extended therapy did not result in a significant increase in complications or hospital readmissions. These findings suggest the importance of careful patient selection and monitoring for OPAT, potentially guiding more efficient and targeted healthcare practices.

**Figure:**